# Analysis of the Ki-67 index in the vaginal epithelium of castrated rats treated with tamoxifen

**DOI:** 10.6061/clinics/2016(02)07

**Published:** 2016-02

**Authors:** Afif Rieth Nery-Aguiar, Yousef Qathaf Aguiar, Airton Mendes Conde Júnior, Airlane Pereira Alencar, Cleciton Braga Tavares, Pedro Vitor Lopes-Costa, Afonso Celso Nazário, Benedito Borges da Silva

**Affiliations:** I Universidade Federal do Piauí, Departamento de Ginecologia; II Morphology, Teresina/, PI, Brazil; III Universidade de São Paulo, Departamento de Estatísticas, São Paulo/, SP, Brazil; IV Universidade Federal de São Paulo, Departamento de Mastologia, São Paulo/, SP, Brazil

**Keywords:** Tamoxifen, Rat, Vagina, Proliferation, Ki-67

## Abstract

**OBJECTIVES:**

Vaginal atrophy and breast cancer are common conditions in postmenopausal women and tamoxifen is the standard endocrine treatment for hormone-sensitive tumors. The present study aimed to assess the effect of tamoxifen on Ki-67 protein expression in the vaginal epithelium of castrated rats.

**MATERIAL AND METHODS:**

Forty Wistar-Hannover adult, virgin, castrated rats were randomly divided into two groups, group I (control, n=20) and group II (tamoxifen, n=20), receiving 0.5 ml of propylene glycol and 250 µg of tamoxifen diluted in 0.5 ml of propylene glycol, respectively, daily by gavage for 30 days. On the 31st day, the rats were euthanized and their vaginas were removed and fixed in 10% buffered formalin for the immunohistochemical study of Ki-67 protein expression. Data were analyzed by the Levene and Student's t tests (*p*<0.05).

**RESULTS:**

The mean index of Ki-67 expression in the rat vagina of groups I and II was 4.04±0.96 and 26.86±2.19, respectively (*p*<0.001).

**CONCLUSIONS:**

According to the results of the present study, tamoxifen, at the dose and treatment length used, induced a significant increase in the cell proliferation of the vaginal mucosa in castrated rats, as evaluated by Ki-67 protein expression.

## INTRODUCTION

The number of postmenopausal women worldwide has increased due to improved access to healthcare services, better living conditions and increased life expectancy. Women spend approximately one-third of their lifetime in the postmenopausal period and may become vulnerable to chronic degenerative diseases such as breast cancer 1. Breast cancer is the most common malignancy among women, particularly in the postmenopausal period. In Brazil, breast cancer is an important cause of cancer-related death, with approximately 57,120 new cases of breast cancer and 13,345 deaths from this disease estimated for the year 2014 2,3,4. However, the survival of women with breast cancer has increased in the last few years, especially due to early diagnosis and to modes of systemic treatment. Endocrine treatment is the main treatment for women with estrogen receptor-positive breast cancer 5. Tamoxifen is a first-generation selective estrogen receptor modulator (SERM) approved by the US Food and Drug Administration (FDA) for primary chemotherapy and adjuvant treatment of women with breast cancer 6.

The mechanism of action of tamoxifen has not been fully elucidated. However, because it interacts particularly with estrogen receptors, tamoxifen may have an effect on symptoms due to hypoestrogenism, such as vaginal atrophy and dyspareunia, which are common in postmenopausal women with breast cancer 7,8,9,10,11. The vaginal mucosal epithelium has both alpha and beta estrogen receptors, with decreasing beta receptor levels after menopause 10. As shown by some authors, tamoxifen exerts both estrogenic and antiestrogenic effects in the vaginal epithelium of castrated rats 12. Nevertheless, other authors, by using indirect methods such as vaginal cytology, have shown that the drug exerts an estrogenic action on the vaginal mucosa of postmenopausal women 13,14,15. The study of drug effects on the vaginal tissue of postmenopausal women is not possible due to ethical reasons. As few studies have investigated the direct effect of tamoxifen on the vaginal epithelium of castrated rats, the present study was conducted.

## MATERIAL AND METHODS

### Animals

This study was approved by the Animal Experimentation Ethics Committee (AEEC) of the *Universidade Federal do Piauí (UFPI)*, complying with the guidelines of the Brazilian College of Animal Experimentation (COBEA). Forty Wistar-Hannover virgin female rats obtained from the animal laboratory of the School of Veterinary Science at UFPI were used. The animals were housed inside a plastic cage with a metallic lid. Five animals per cage were kept since birth with free access to filtered water and rodent chow (SUPPRALAB^®^, São Paulo, Brazil). Ambient conditions were controlled by a 12-hour light/dark cycle, with the lights switched on from 7 a.m. to 7 p.m. Air conditioning maintained the temperature between 20 and 25°C. The animals were randomly divided into two groups: group I (control, n=20) and group II (tamoxifen, n=20). At 90 days of age, the rats in groups I and II weighing an average of 231.5±20.85 g and 231.2±21.74 g (*p*=0.9647), respectively, were castrated to eliminate gonadal sex steroid production through a dorsal paravertebral incision under anesthesia with 60 mg/kg ketamine and 4 mg/kg midazolam. Twenty-one days later, cytologic examination of Papanicolaou-stained vaginal smears was performed to characterize a decreased estrogen status, which was confirmed by the absence of cell maturation and a predominance of basal cells in all the animals. Group I rats (control) began to receive 0.5 ml/animal/day of propylene glycol (placebo) and Group II rats (experimental) received 250 μg/animal/day of tamoxifen citrate diluted in 0.5 ml of propylene glycol. The placebo and tamoxifen were continuously administered for 30 days, both at the same hour of the day, using a metal tube appropriate for gavage. On the 31st day, the rats from both groups were sacrificed and their vaginas were removed through a longitudinal abdominal incision for histologic and immunohistochemical studies.

### Ethics Approval

This study was approved by the Animal Experimentation Ethics Committee (AEEC) of the Universidade Federal do Piauí (UFPI).

### Ki-67 Immunohistochemistry

For the immunohistochemical study of Ki-67 expression, tissue specimens were fixed in 10% buffered formalin for a 12-24-hour period and then cut into 5-µm-thick sections. The sections were processed and stained with hematoxylin-eosin, deparaffinized in xylene for 5 minutes, dehydrated in absolute ethanol and washed in buffered saline solution at pH 7.4 for 5 minutes 16. Endogenous peroxidase activity was blocked with 3% hydrogen peroxide (H_2_0_2_) diluted in a buffered solution for 5 minutes. After epitope retrieval, the tissue samples were incubated with primary mouse anti-Ki-67 monoclonal antibody (clone MIB-5/Dako/1:100) in a refrigerator at approximately 4°C overnight. Subsequently, the samples were washed with buffered saline solution and incubated for 45 minutes in the NovoLink Polymer detection system. Color development was accomplished by incubating all the tissue samples in 3-3-diaminobenzidine tetrahydrochloride solution at a concentration of 1 mg/ml in a Tris-buffered solution, with a hydrogen peroxide solution used as a substrate, for 5 minutes. Subsequently, the tissues were counterstained with Harris' hematoxylin or methyl green for 5 minutes, followed by dehydration in ethanol and xylene baths. After the procedure, the slides were examined to quantify the results. Cells expressing Ki-67 protein were identified by the dark brown color of their nuclei.

### Quantitative method

Ki-67 expression was quantified by two observers who were blinded to the group identification. Stained images were assessed by using a Nikon E400 light microscope connected to a Samsung color digital video camera, model SCC-131. The video camera captured and transmitted the image to a computer equipped with ImageLab^®^ software, version 2.3, which was developed by *Softium Informática Ltda* (São Paulo, Brazil) for image analysis. To evaluate Ki-67 expression, 500 vaginal epithelial cells were counted on each slide whether or not the cells were stained with anti-Ki-67 MIB-5 antibody. A total magnification of ×400 was used to examine the slides. Cell counting was initiated at the site of greatest Ki-67 antigen expression. In each case, the percentage of stained cells was obtained by the ratio between the number of cells with stained nuclei and the total number of cells multiplied by 100.

### Statistical Analysis

To evaluate group homogeneity relative to weight, Student's t test was used for independent samples. Variance analysis of the percentages of nuclei stained with anti-Ki-67 was conducted using the Levene and Student's tests 17. The significance level was set at *p*<0.05.

## RESULTS

**Figure 1-:**
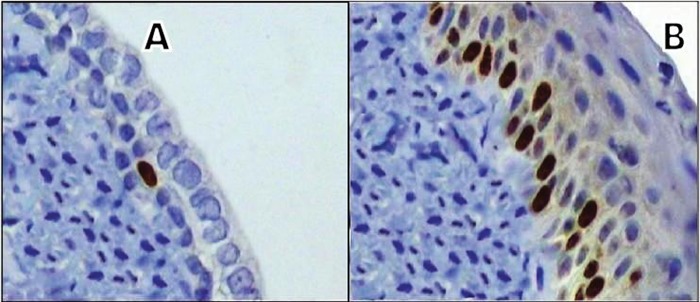
Photomicrograph of a histological section of the vaginal epithelium of castrated rats showing a greater concentration of Ki-67-stained nuclei in the experimental group (tamoxifen) **(B)** compared with the control group **(A)** (original magnification x400).

**Figure 2:**
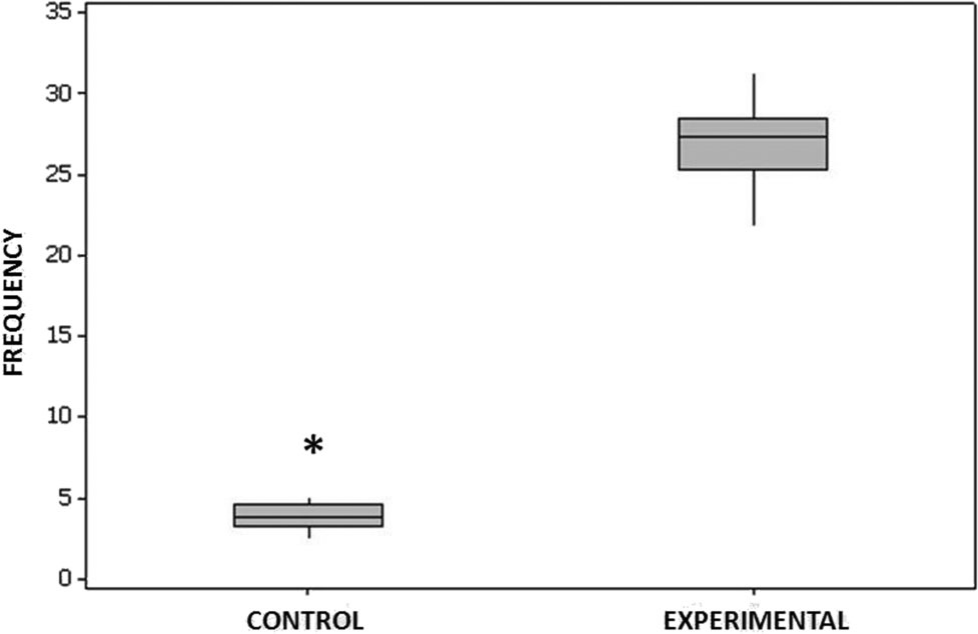
The median of Ki-67-stained nuclei in the vaginal mucosa of animals in the control and tamoxifen groups, as illustrated by the line in boldface.

By light microscopy, cytologic examination of the vaginal epithelial cells of control group animals showed the absence of cell maturation and a predominance of basal cells, while tamoxifen-treated animals showed predominant cell maturation. Compared with control group animals, castrated female rats treated with tamoxifen showed a higher concentration of nuclei stained with anti-Ki-67 (Figure 1). The mean percentage of MIB-5-stained nuclei in the vaginal mucosa of group I (control) and group II (tamoxifen) rats was 4.04±0.96 and 26.86±2.19, respectively (*p*<0.001) (Table 1). The boxplot illustrates the median Ki-67 expression in the vaginal mucosal cells of animals from the control and tamoxifen groups (Figure 2).

## DISCUSSION

In the present study, tamoxifen at a dose of 250 μg/animal/day administered for 30 days significantly increased proliferation of the vaginal mucosa in castrated rats, as evaluated by Ki-67 protein expression. The animals received tamoxifen orally (gavage) to mimic the administration route used in women. Furthermore, according to several authors that have studied different routes of administration, the oral route led to higher serum and tissue levels of tamoxifen, thereby increasing the bioavailability of tamoxifen 18,19. Tamoxifen was diluted in propylene glycol, a neutral, colorless and odorless vehicle, allowing for the dilution and administration of predefined doses 20,21.

However, the tamoxifen dose in animal experimental studies is not easy to standardize because rats have a faster metabolism than humans. Equal-weight doses may not reproduce the serum levels and effects of tamoxifen in women 22. Dos Santos et al. 21, who studied the morphometry of the urethra in castrated rats given a 250 µg/day tamoxifen dose for 30 days, observed that tamoxifen was capable of increasing the uterine weight and the thickness of the distal urethral epithelium. Thus, we used a dose of 250 µg/animal/day in this study because it could mimic the effects of tamoxifen on women undergoing breast cancer treatment 22.

Friedrich et al. 13 studied the influence of tamoxifen on vaginal epithelial maturation in postmenopausal women and demonstrated an apparent increase in vaginal epithelial maturation. In another study, Friedrich et al. 14 investigated the effects of tamoxifen on vaginal and cervical epithelial proliferation in postmenopausal women with breast cancer and found an apparent increase not only in the incidence of metaplasia and hyperplasia of endocervical cells but also in vaginal epithelial maturation. According to Bertolissi et al. 15, tamoxifen exerts an early and persistent estrogenic effect on the vaginal epithelium during the first year of therapy in postmenopausal women with breast cancer.

Nevertheless, Berg and Dunfee 23 showed that postmenopausal women with high levels of squamous epithelial cell maturation were 15 times more likely than women in the same age group exhibiting an atrophic pattern to have coexistent endometrial adenocarcinoma because tamoxifen acts in accordance with circulating estrogen levels. Therefore, when estrogen levels are low, tamoxifen behaves as an agonist and may cause endometrial cancer. In contrast, during the premenopausal period, when estrogen levels are higher, tamoxifen appears to have an antiestrogenic action in this tissue 24. Thus, the agonist or antagonist action of SERMs in the endometrium may depend on circulating estrogen levels 25,26.

The results of the present study indicate that tamoxifen, at the dose and length of treatment used in this study, induced a significant increase in cell proliferation of the vaginal mucosa in castrated rats, as evaluated by Ki-67 protein expression. Thus, the assessment of cell proliferation in the vagina of castrated rats, used to as a mimic of postmenopausal women, revealed that tamoxifen displayed partially estrogenic action on the vaginal mucosa of these animals.

## AUTHORS CONTRIBUTION

Nery Aguiar AR and Aguiar YQ contributed to the data collection and to the manuscript writing. Alencar AP contributed to the statistical analysis. Conde Jr AM, Lopes-Costa PV and Tavares CB contributed to the data collection. da Silva BB conceived and planned the study and contributed to the data collection, to the manuscript writing and to conducting this study.

## Figures and Tables

**Table 1:** Mean percentage of Ki-67-stanined nuclei in the vaginal mucosa of groups I (control) and II (tamoxifen).

GROUP	N	AVERAGE	SE	SD	MINIMUM	MEDIAN	MAXIMUM
**I**	20	4.04	0.21	0.96	2.51	3.86	6.69
**II**	20	26.86*	0.49	2.19	21.77	27.38	31.23

**<?ENTCHAR ast?>:** p<0.001
